# Self-collection for primary HPV screening using dry swabs: a review of clinical performance, laboratory considerations, and patient preferences

**DOI:** 10.1128/jcm.00767-24

**Published:** 2025-10-21

**Authors:** Mondraya F. Howard, Ria C. Fyffe-Freil, Christina C. Pierre, Henrietta O. Maku, Stefania Di Costanzo, Thomas S. Lorey, Dina N. Greene

**Affiliations:** 1 Department of Pathology and Laboratory Medicine, Penn Medicine Lancaster General Health 209639 , Lancaster, Pennsylvania, USA; 2 Department of Pathology, Microbiology and Immunology, University of Nebraska Medical Center 12284, Omaha, Nebraska, USA; 3 Department of Pathology and Laboratory Medicine, Perelman School of Medicine, University of Pennsylvania 6572, Philadelphia, Pennsylvania, USA; 4 Department of Pathology and Genomics, Houston Methodist Hospital 23534 , Houston, Texas, USA; 5 Copan Italia S.p.A 165421 , Brescia, Italy; 6 Independent Researcher, Oakland, California, USA; 7 Department of Laboratory Medicine and Pathology, University of Washington 189576, Seattle, Washington, USA; 8 Evergreene Labs, Burien, Washington, USA; Vanderbilt University Medical Center, Nashville, Tennessee, USA

**Keywords:** primary screening, HPV, cervical cancer, vaginal swab, pre-analytical, dry swab, self-collection

## Abstract

For cervical cancer screening, testing for high-risk human papillomavirus (hrHPV) detects more high-grade precancerous lesions, has a higher negative predictive value, and requires fewer lifetime screenings compared to cytology. As a result, both the US Preventive Services Task Force and the World Health Organization have endorsed hrHPV testing as the preferred screening method. Despite the utility, there are significant implementation challenges to adopting hrHPV primary screening across patients, providers, and institutions that must be addressed to ensure its widespread effectiveness. Here, we take a laboratory-centric approach to reviewing hrHPV primary screening, including discussion of specimen types and collection methods. For user experience, clinical and analytical validations, we focused on self-collected vaginal swab specimens stored dry during transport. Our analysis indicates that clinical laboratories should do their part to engage with institutional and clinical leadership to validate and promote the use of vaginal self-sampling for cervical cancer screening options within and outside the clinic. This work highlights the multiple studies that have validated dry swab collection as a simplified and high-quality method for hrHPV detection.

## INTRODUCTION

Reducing the incidence of cervical cancer relies on preventative care, specifically access to and uptake of vaccination and screening (1). Benign cervical lesions, such as condyloma acuminata, genital warts, and low-grade squamous intraepithelial lesions are caused by low-risk human papillomavirus genotypes (e.g., 6 and 11). These are distinct from high-risk human papillomavirus (hrHPV) types (e.g., 16, 18, 31, 33, and 45) that are implicated in high-grade squamous intraepithelial lesions, precancer, and cervical cancer. Presently, hrHPV primary screening, or the detection of hrHPV nucleic acid sequences from vaginal fluid or cervical cells, has proven to be the most effective, practical, and efficient method, and it has been endorsed as the primary approach for cervical cancer screening by the World Health Organization (2) (Table 1). In alignment with this, most societal and governmental guidelines include an option for hrHPV primary screening in their cervical cancer prevention recommendations (Table 2). hrHPV testing is more effective at detecting high-grade pre-cancerous lesions, has a higher negative predictive value, and requires fewer lifetime screens compared to cytology. Additionally, as increased uptake of HPV vaccination continues to reduce the prevalence of hrHPV types and cervical lesions, Pap testing will become less effective and less efficient, while hrHPV testing will maintain superior performance characteristics (1).

**TABLE 1:** Definitions of commonly used terms

Term	Definition
hrHPV	High-risk HPV includes types 16, 18, 31, 33, 35, 39, 45, 51, 52, 56, 58, and 59. Types 16 and 18 are *most commonly* associated with cervical cancer.
lrHPV	Low-risk HPV does not cause cancer but can be associated with warts (most often types 6 and 11). Not indicated for cervical cancer screening programs.
Remote collection	Refers to the self-collection of vaginal swab specimens outside of the clinic setting. Also known as *home collection*, *self-collection*, *self-sampling*, or *out-of-clinic collection*.
Co-testing	Both Pap test and hrHPV testing are performed at initial screening. A cervical cytology sample is visually examined for abnormal/atypical cellular morphology and analyzed by nucleic acid amplification for HPV detection.
HPV primary screening	High-risk HPV testing is performed as initial screening. Clinician-collected cervical cells or self-collected vaginal samples are analyzed by nucleic acid amplification for HPV detection.
High-grade precancerous lesions	Refers to a high-grade squamous intraepithelial lesion on Pap test cytology Cervical intraepithelial neoplasia grades 2 and 3 (CIN 2 and CIN 3), moderate and severe dysplasia, and carcinoma *in situ* on cervical biopsy. Associated with hrHPV infection *Note: due to a tendency for natural regression, CIN2 in women of childbearing age is managed by observation like CIN1*.
Transition zone	Squamocolumnar junction; most common site of HPV infection in the cervix. Important for morphology when evaluating cervical cells, but it is not necessary to collect from this area for HPV detection.

**TABLE 2:** Comparison of screening algorithms^a^

Organization	Age specific recommendation			
	Ages 21-29	Ages 30-65		
USPSTF (2018)	Every 3 years with cytology only	Every 3 years with cytology only AND every 5 years with hrHPV testing; OR	Co-testing every 5 years	
ACOG (2021,reaffirmed 2024)	Every 3 years with cytology only	Every 3 years with cytology only; OR	Every 5 years with hrHPV testing; OR	Co-testing every 5 years
ACS (2020)	Ages 25-65			
	Every 5 years with hrHPV testing (If not available, then co-testing every 5 years or cytology only every 3 years is recommended)			
WHO (2021)	Ages 30-49			
	Every 5-10 years Screen-and-treat (hrHPV testing only, treat if positive); OR Screen, triage and treat (hrHPV testing, then if positive, cytology or equivalent)			
EC-CvC (2025)	Ages 25-29		Ages 30-64	
	hrHPV testing as primary screen with effective triage strategies for HPV-positive individuals		hrHPV testing as primary screen	
NCSP (2025)	Ages 25-74			
	Every 5 years with hrHPV testing (including partial genotype and cytology triage)			

^*a*^ All screening guidelines apply to women and those with a cervix. The United States Preventive Services Task Force (USPSTF) guidelines are undergoing revision in 2025. The WHO has different recommendations for women living with HIV, not included here. The American College of Obstetricians and Gynecologists (ACOG), American Cancer Society (ACS), World Health Organization (WHO), and National Cervical Screening Program (Australia; NCSP).

Cytology was the first and only laboratory method used to screen the cervix for pre-cancerous lesions until the early 2000s when detection of hrHPV DNA was integrated into the screening pathway to triage equivocal cytology results (1). Over the next decade, this evolved into co-testing, whereby a cervical cytology sample is evaluated visually for abnormal/atypical cellular morphology and by nucleic acid amplification for hrHPV detection. Co-testing remains a common screening pathway, despite the clinical validation of hrHPV primary screening indicating that cytology should be used selectively, or more specifically, as a triage method when certain hrHPV genotypes are detected (3, 4). Regardless of the screening methods employed, the recommended follow-up based on abnormal results will be identical (5).

Here, we take a laboratory-centric approach to reviewing HPV primary screening, defining the challenges to implementation and describing specimen types and collection methods. For user experience, clinical and analytical validations, we focused on self-collected vaginal swab specimens stored dry during transport. Our analysis indicates that clinical laboratories should validate and promote the use of vaginal self-sampling with dry transport for cervical cancer screening programs within and outside the clinic.

## SPECIMEN TYPES FOR hrHPV PRIMARY SCREENING

Cervical samples collected by clinicians remain the reference standard for the detection of hrHPV nucleic acid sequences due to reliable sampling of the transformation zone, the area that HPV most commonly infects, and compatibility with both cytology and molecular assays. These specimens are typically obtained using a cervical brush and preserved in alcohol-based cytology media such as PreservCyt (Hologic) or SurePath (BD), which permit concurrent cytologic triage and molecular detection (6, 7). However, self-collection—particularly of vaginal specimens—has gained widespread attention as an alternative, especially given increasing evidence for non-inferior clinical sensitivity compared to clinician-collected samples when paired with validated amplification-based HPV assays (8–10). Vaginal samples are inadequate for the collection of cervical cells for cytology testing. Accordingly, for individuals undergoing primary HPV testing with a vaginal self-collected specimen, only those who test HPV positive—approximately 15%—will require a pelvic examination to collect cervical cells for triage testing. By comparison, all individuals undergoing co-testing require a pelvic exam so the clinician can collect a cervical sample for both HPV and cytology testing.

Several vaginal self-collection devices are in use or under evaluation, including flocked swabs (e.g., Copan FLOQSwabs), brush-like devices (e.g., Evalyn Brush, Viba-Brush, and Qvintip), foam-like devices (e.g., Teal Wand), and tampon-based samplers (Table 3). Among these, Evalyn Brush and FLOQSwabs have been most extensively evaluated within validation frameworks, such as VALHUDES, and have shown high clinical sensitivity (≥89%) for high-grade cervical intraepithelial neoplasia detection when used with amplification-based assays, including Roche cobas, BD Onclarity, Abbott RealTime HPV, and Seegene Anyplex (10–13). In contrast, devices such as Qvintip and tampon-based methods have demonstrated more variable sensitivity, falling below non-inferiority thresholds in some studies (14, 15). The most widely adopted devices among the ones listed are Rovers Evalyn Brush and Copan Self Vaginal FLOQSwabs. The Evalyn Brush is a dry brush used for cervicovaginal self-sampling containing flexible bristles, an inserter with stopper wings for correct depth of insertion, and a retractable plunger that pushes the brush into the cervicovaginal junction. The FLOQSwabs is a dry, sterile, flocked swab often used for patient-collected vaginal specimens. The FLOQSwabs shaft includes a red mark to indicate where to hold the swab during specimen collection and is contained in a tube used for sample storage and transport to the laboratory.

**TABLE 3:** Key features of common self-sampling devices in use or under evaluation

Device type	Device name (manufacturer)	Material and features
Vaginal swabs	FLOQSwabs (Copan)	Nylon-flocked swab, red mark guide on swab shaft to indicate correct insertion depth FDA-approved for self-collection with Roche Cobas and BD Onclarity HPV assays in healthcare setting, 2024
	Qvintip (Aprovix)	Plastic head with grooves for vaginal fluid collection; head is removed and transported in a separate tube
Brush	Evalyn Brush (Rovers Medical Devices)	Flexible, low-density polyethylene (LDPE) bristles, an inserter with stopper wings for correct depth, and plunger mechanisms for bristle extension/retraction FDA-approved for self-collection with Roche Cobas HPV assay in healthcare setting, 2024
	Viba Brush (Rovers Medical Devices)	LDPE bristles on sampling head attached to shaft without stopper guide; sampling head is removable for transport
	HerSwab (Eve Medical^*a*^)	Curved tip with retractable brush for cervicovaginal cell collection and plastic applicator handle
Sponge	Teal Wand (Teal Heal)	Retractable device with soft sponge material for cell collection; sponge is removed for transport FDA-approved for at-home self-collection, 2025

^*a*^ As of this report, the status of the manufacturer is no longer an established business.

A key determinant of performance for self-collected specimens is the resuspension buffer and volume. Clinician-collected cervical specimens are preserved in 10–20 mL of PreservCyt or SurePath, but this volume may dilute analyte concentration in vaginal samples, which often yield lower cell counts and viral loads (16). Studies have shown improved assay sensitivity when self-collected swab specimens are resuspended in ≤5 mL of media, particularly lytic media such as BD diluent, eNat, or MSwab that optimize nucleic acid recovery (17–20). These media allow for stable preservation of HPV nucleic acids for up to 4 weeks, even at ambient or elevated temperatures, which is essential for decentralized screening programs in low-resource settings (18, 20).

Urine sampling, especially first-void urine, is also under investigation as a non-invasive alternative. While studies have shown good clinical sensitivity using devices such as Colli-Pee (Novosanis), urine samples are more biologically heterogeneous and require immediate stabilization to prevent degradation (21–23). There is limited evidence on the long-term stability of HPV nucleic acid sequences in urine, especially during extended ambient-temperature transport (21). This presents a constraint for implementation in programs relying on mailing or community-based outreach, where sample integrity during transport may be difficult to guarantee (22, 23).

Dry flocked swab specimens can be shipped without buffer, minimizing leakage risk and biohazard concerns. The specimens can be resuspended under controlled laboratory conditions, enhancing standardization of pre-analytical workflows. HPV DNA has been shown to remain stable on self-collected dry swabs even when challenged with extreme temperatures (24). Collectively, these operational advantages—along with performance data—highlight the relevance of dry swabs as a focus of implementation research. Here, we focus on dry swab-based HPV self-sampling to support assay validation and optimized workflow development for broader population-level screening.

## SELF-SAMPLING IN REMOTE OR NON-CLINICAL SETTINGS INCREASES CERVICAL CANCER SCREENING UPTAKE

Cervical cancer screening using vaginal specimens self-collected outside the clinic was examined in five studies (25–29). Screening uptake was higher for vaginal specimens self-collected outside the clinic compared to clinician-collected cervical specimens across three studies of under-screened or never-screened cohorts (26–28). Screening uptake ranged from 22.5% to 93% for home-collected samples and 11.7% to 56% for clinic-collected samples. In a randomized trial of 400 Nigerian participants, screening uptake was 93% (185/200) in the group randomized to at-home self-collection versus 56% (113/200) randomized to the clinician-collected group (28). The return rate of specimens was 80% in a study of 500 Italian participants who were invited by phone to complete home self-sampling instead of routine in-clinic cervical cancer screening (25), and 77.5% in a study of 400 Québécois participants who were recruited by radio, social media, and television advertising to complete home self-sampling. Of note, in the aforementioned studies, participants “opted in” prior to being mailed self-sampling kits (25, 28).

In studies that employed an “opt-out” approach, lower screening uptake was observed in both outside and within clinic cohorts, with specimens collected outside the clinic achieving higher uptake (26, 27). Sultana et al. (27) identified participants residing in Victoria, Australia, who were never screened or past due for cervical cancer screening. Participants were randomized to home vaginal self-sampling and Pap test groups. The self-sampling group was given the option to “opt out” prior to being mailed self-sampling kits. Screening uptake was 20.3% for the self-sampling group versus 6% for the Pap test group. Haguenoer et al. (26) identified participants from a French University Hospital Cancer screening database who were due for screening and did not respond to mailed invitations to obtain in-person screening. Participants (*n* = 6,000) were randomized to receive a recall letter for in-person screening, a vaginal dry-swab self-sampling kit, or no intervention. After 12 months, the uptake rates for the in-person recall, self-sampling, and no intervention groups were 13.8%, 24.4%, and 12.4%, respectively. These studies unequivocally demonstrate that home self-sampling can increase cervical cancer screening uptake; however, studies that utilize “opt-in” approaches may overestimate uptake rates that can be anticipated in general population screening.

## PATIENTS CONSIDER VAGINAL SELF-SAMPLING FAVORABLE FOR CERVICAL CANCER SCREENING

The user acceptability of vaginal self-collection was evaluated in 12 studies (28–39), of which eight examined whether participants preferred self-collection of vaginal swab specimens versus clinician-collected cervical samples (28, 29, 31–35, 39). In seven out of eight studies, a majority (≥83%) of participants preferred self-sampling over clinician-collected samples (28, 29, 31–33, 35, 36). Ease of use, comfort of sampling, convenience, lack of embarrassment, flexibility in timing and/or place of collection, availability of screening, and time savings were cited as advantages or reasons for preference of self-collection. In a Malaysian study of 725 healthy participants, barriers to completing prior Pap testing included inconvenience, fear of pain, embarrassment, and lack of awareness (33). Furthermore, participant preference for self-collection was associated with full-time employment and higher income, while preference for clinician collection was associated with a history of prior Pap tests.

About 30% of participants preferred self-collection of vaginal specimens over physician-collected cervical samples in a cross-sectional study of 399 Kenyan sex workers (34). In this study, participants expressed concern about proper self-collection and pain during self-sampling. In another study of 310 Norwegian patients referred for conization of premalignant cervical lesions, with confirmed cervical carcinoma or suspicion of cervical carcinoma, 84% had more confidence in physician-collected samples, although 88% considered self-collection a good alternative (36). Furthermore, when asked what screening method they would opt for in the future, 81% favored self-collection. In a multi-center cross-sectional study that compared clinician-collected, dry swab vaginal self-collected, and liquid medium vaginal self-collected cervical cancer screening in a cohort of 722 French participants (30), 104 (14.4%) reported that they encountered difficulties during the self-sampling procedure, including uncertainty in where or how to introduce the swab or the required depth of insertion. About 1 in 11 women (8.9%) reported pain or discomfort, and a small percentage (1.8%) asked for clinician assistance during self-collection. Notably, the study did not report on participants’ impression of the clinician collection process; however, fear of pain and embarrassment is a well-documented barrier to conventional clinician-collected cervical cancer screening specimens (30, 40).

Preference for maintaining the swab dry versus wet following specimen collection was variable, with a marginal preference toward dry transport. In a crossover trial of 120 women presenting to a Swiss colposcopy clinic, participants were instructed to self-collect two consecutive vaginal samples, after which one swab was placed into an empty collection tube for dry transport and one swab was placed into a collection tube containing transport buffer (37). Self-sampling was viewed favorably by 96.4% of participants, and most women (74%) viewed dry and wet swab self-collection as equally reliable. While most women perceived no difference in complexity between self-collection sampling methods, 15 viewed wet swab collection as more complex, and six viewed dry swab self-collection as more complex. Vaginal self-collection was marginally preferred over urine collection for hrHPV testing in a randomized cohort of 454 women who completed self-sampling at a London colposcopy clinic (39). Participants, however, reported the greatest confidence in correct collection for urine compared with vaginal swab collections. In a subset of women who completed dry and wet swab self-collection (*n* = 222), 71.4% preferred a dry swab. Notably, one wet and three different dry swabs were compared in this study, and one of the dry swabs was ranked as the most difficult to use.

Together, these studies demonstrate a largely positive sentiment toward self-collection of vaginal specimens as a cervical cancer screening method. Optimization of collection instructions, education on the reliability of cervical cancer screening using self-collection methods, and availability of assistance with a hotline, app, or web-based chat may mitigate residual concerns associated with self-sampling, particularly when performed outside the clinic.

## HIGH RATES OF APPROPRIATE FOLLOW-UP OF hrHPV-POSITIVE RESULTS OBTAINED VIA SELF-SAMPLING

Follow-up of positive hrHPV test results with Pap tests and/or colposcopy is critical to cervical cancer prevention and has been investigated as a potential barrier to implementation of home-based self-collection. Patient compliance with appropriate follow-up for hrHPV-positive test results from self-collected samples has been reported to be 75%–97% (26, 27, 29). In a French study, adherence to follow-up was 80.8% in participants randomized to the no intervention or recall group with abnormal Pap smear screening results (26). This is similar to the baseline follow-up rate (61.7%–80.3%) observed in a New Mexico state-wide surveillance program evaluating guideline-adherent follow-up after abnormal co-testing results (41). In a Greek study that aggregated data from mid-wife-assisted clinic and home-collected samples, the follow-up compliance rate was 72% (31). Barriers to completion of appropriate clinical follow-up included patient refusal (27, 31), pregnancy (31), and difficulty contacting participants (27, 31).

## SELF-COLLECTED VAGINAL SPECIMENS TRANSPORTED DRY ARE CLINICALLY EQUIVALENT TO CERVICAL SAMPLES FOR THE DETECTION OF hrHPV

A meta-analysis of 56 accuracy studies showed that hrHPV detection on self-collected specimens is equivalent to clinician-collected samples for the detection of cervical precancers (8). These studies largely included self-collected specimens transported in cell preservative or virological transport media. Studies that utilized dry swabs (*n* = 7) had relative accuracy estimates for detecting high-grade precancerous lesions of 0.96 (95% CI: 0.90–1.02) for relative sensitivity and 1.01 (95% CI: 0.94–1.10) for relative specificity, suggesting a slightly better specificity when collected as dry samples. However, neither is statistically significant at the 95% CI. Pooled overall agreement (88.7%) was determined for 26 studies, of which three studies included self-collected vaginal swab specimens that remained dry for transport (42).

In a literature search using the terms “HPV AND dry swab,” 21 studies (12, 13, 15, 23, 30, 32, 34, 36, 38, 39, 43–53) were identified, and 14 were cross-sectional diagnostic studies using assays with FDA or CE approval for primary hrHPV screening and compared clinical sensitivity between self-collected vaginal swab specimens and clinician-collected cervical specimens (12, 13, 34, 36, 38, 39, 45–51, 54). Overall, these studies indicated that the two specimen types are comparable; detection of hrHPV in self-collected dry swab or dry brush samples exhibited >81% sensitivity and >81% concordance compared to cervical samples. Most studies (*n* = 11) occurred in a follow-up setting where participants were referred for colposcopy (12, 13, 34, 36–39, 42, 47, 54, 55) or treatment for precancerous lesions (36); two studies included participants from a routine screening or low-risk population (50, 51), whereas another study enrolled participants from a high-risk cohort of HIV-infected individuals (34). High-risk HPV detection was more prevalent in the follow-up and high-risk populations (42%–90%) (12, 13, 34, 36, 37, 39, 46, 47, 49, 55) than in individuals enrolled from routine screening populations (10%–25%) (30, 47, 50, 51). Performance of vaginal self-samples collected with swab or dry brush in a colposcopy setting exhibited at least 86% concordance for hrHPV detection with paired cervical samples and >83% clinical sensitivity for detecting high-grade precancerous lesions when used with amplification-based assays, including BD Onclarity, Alinity m hrHPV, cobas HPV, and Xpert HPV (39, 47, 49, 54). While studies conducted in referral populations enable higher powered measurements of sensitivity due to the higher prevalence of hrHPV, this is not the intended use population for hrHPV self-collection.

There were two studies that compared self- and clinician-collected specimens for hrHPV detection in routine screening populations (30, 51). In a Dutch screening program, where cervical smears were the primary screening method, 2,049 participants were provided a self-sampling kit at the time of their appointment, which included a dry brush sampling device to self-collect vaginal specimens following collection of cervical samples by clinicians (51). The results illustrated 96.8% agreement between self-collected and physician-collected specimens using the cobas HPV assay. In a French study of 722 participants due for routine screening Pap test, 91.7% concordance was reported between self-collected and clinician-collected samples in detecting hrHPV. An HPV genotyping assay that detects 32 hrHPV types was used to assess clinical performance. Vaginal self-collected specimens using a dry nylon-flocked swab displayed 88.7% sensitivity and 92.5% specificity for detecting hrHPV compared to cervical samples (30). Together, these studies, while limited, indicate that self-sampling in a routine screening population has a high level of agreement with provider-collected cervical samples. The reported diagnostic accuracy (88% sensitivity and >92% specificity) for hrHPV detection in the intended use population for self-sampling supports the use of dry swabs as a method to detect hrHPV infection and identify individuals for targeted follow-up.

Calculated sensitivity and specificity in all studies use the cervical specimen as the gold standard, which is appropriate, but can be misleading because self-collected vaginal swabs have been shown to detect more samples with hrHPV relative to the paired cervical specimen across several studies (13, 24, 30, 34, 43, 48, 50, 51, 53, 55, 56). In a cohort of 303 Australian participants scheduled for colposcopy, Saville et al. (13) evaluated the performance of six clinically validated HPV assays with self-collected vaginal specimens using a flocked swab compared to cervical specimens. Self-collected dry swabs detected more hrHPV compared to physician-collected cervical samples, with an absolute percent increase of 4.2%–11.2% depending on the HPV assay used. Similar rates of hrHPV detection in self-samples were observed in other studies, regardless of patient population (follow-up versus routine screening). Paired self-collected vaginal and provider-collected cervical samples were obtained from 144 participants at follow-up colposcopy visits (24). High-risk HPV was detected in 62.5% (90/144) of vaginal self-collected nylon-flocked swabs compared to 59% (85/144) of cervical samples. In over 2,000 participants of a Dutch screening program, hrHPV prevalence was shown to be 8% in cervical samples and 10% in vaginal self-collected samples (51). Moreover, self-collected dry specimens generally displayed increased sensitivity for clinically relevant hrHPV than cervical samples. Swiss participants (*n* = 150) referred for colposcopy performed vaginal self-sampling with a cotton swab prior to their gynecologic examination. High-grade precancerous lesions were observed in biopsies of 36.5% (27/74) of participants. The hrHPV-positivity rate in self-samples collected from this subset was 84.2% compared to 73.1% of cervical samples (55). In a general screening cohort of 722 participants, 79 individuals had abnormal cytology results; 66% of vaginal self-collected samples were positive for hrHPV in this cytology subset, whereas cervical sampling produced HPV-positive results in 64.6% of specimens (30). High-grade precancerous lesions were detected in <1% of the screening population (6/722), and vaginal self-sampling detected hrHPV in all cases; cervical sampling missed one case. Together, these results suggest that self-collected vaginal swabs that remain dry for transport perform better in detecting cervical abnormalities associated with hrHPV compared to cervical specimens and are therefore appropriate for clinical use.

Randomized clinical trials assessing the efficacy of direct mailed, at-home self-sampling have shown that this strategy increases participation in cervical cancer screening among under-screened and screening-adherent individuals (27, 31, 57). The nature of randomized studies is to determine overall public health implications and not to compare specimen types, while clinical validation studies can directly ensure specimen type equivalency and further validate at-home collection for cervical cancer screening programs. There were three studies that evaluated remote vaginal self-collection (36, 50, 51). Self-sampling devices evaluated in these studies included the Evalyn Brush and FLOQSwabs. At-home collection was found to have comparable clinical sensitivity to clinician-collected cervical specimens when hrHPV testing was performed with PCR assays, including Anyplex II HPV28, cobas 4800 HPV, and Xpert HPV (36, 50, 51). Clinical performance differed between sampling devices, with the Evalyn Brush showing slightly higher concordance with cervical specimens (>90%) and increased sensitivity for detecting high-grade precancerous lesions (>90%) compared to FLOQSwabs (>87% agreement and >86% sensitivity) for all HPV assays (36). In another study, self-sampling kits were given to 2,049 patients attending appointments for routine cervical smears for collection at home or in the clinic (51). Ninety-three percent of participants elected to perform self-sampling using the Evalyn Brush at home. Self-samples detected 90% of high-grade precancerous lesions found by cervical smear and histology.

## VAGINAL SELF-SAMPLING DEVICES HAVE BEEN PARTIALLY VALIDATED TO ADDRESS BROAD PRE-ANALYTICAL CONCERNS

Optimization of technical parameters that impact the performance of HPV testing on self-collected samples is important to support laboratory validation. In cervical cancer screening programs that offer self-sampling, dry swabs are distributed by mail with subsequent resuspension taking place in the laboratory (58). Sample collection device and resuspension volume were found to have the most influence on the performance of self-collected specimens. Resuspension volumes varied across studies, ranging between 2 and 20 mL (13, 24, 54, 59, 60). A loss of sensitivity was observed when vaginal self-samples were resuspended in 20 mL of PreservCyt, a similar volume used for processing cervical specimens (59), but not when self-samples were transferred into volumes less than 5 mL (13, 24, 54, 60). Studies that utilized cotton-tipped swabs for self-sampling observed high invalid rates (11%–24%) compared to self-sampling with flocked swabs (2%) or cervical samples (3%) (55, 60, 61). Direct comparison of cotton swabs and flocked swabs indicated that flocked swabs achieve better cellular retrieval (61). Cadman et al. (39) conducted a paired comparison of 600 vaginal samples collected with four sampling devices, a dacron-tipped swab transported in specimen transport media (1 mL), and FLOQSwabs, Qvintip, and HerSwab as dry samples resuspended into 8 mL of PreservCyt solution before testing. The number of cells collected by the devices was determined using Ct values for the β-globin internal control using the BD Onclarity HPV assay. FLOQSwabs displayed the greatest cellularity among the vaginal self-collection devices. Furthermore, FLOQSwabs displayed better performance for HPV testing compared to other devices. Together, these studies demonstrate that reduced resuspension volume and using devices designed to maximize cell collection enhance the accuracy and sensitivity of HPV self-sampling.

Despite the implementation of HPV self-sampling in several countries (58), there are limited data available surrounding the analytical stability of dry self-samples, particularly regarding self-collection in non-clinical settings where samples may be exposed to prolonged storage or fluctuating temperatures during transportation to the laboratory. These analytical studies are necessary to guide laboratory validation of HPV tests on self-samples. Qi et al. (24) evaluated how extended ambient dry storage and exposure to extreme temperatures affected the performance of self-collected vaginal swabs for hrHPV detection using the cobas HPV assay. In this study, 144 paired self-collected vaginal swabs and provider-collected cervical samples displayed a high level of agreement (90.3%) for hrHPV detection. Three self-samples (2%) that resulted as invalid (hrHPV-negative, β-globin negative) had an average storage time of 27 days. This invalid rate is higher than the observed rates (<1%) in large randomized controlled trials (57, 62). Time studies of ambient storage indicate that while detection of hrHPV DNA remains stable for at least 30 days (range 4–41 days), β-globin is sensitive to storage time. Additionally, hrHPV detection was not impacted by extreme temperatures (−10°C–40°C), whereas temperature changes may degrade the β-globin internal control (24). Overall, dry self-samples were found to be stable for at least 30 days and unaffected by exposure to temperatures that might be encountered during transit through the mail. In a US-based clinical trial, the median time to screening completion for participants randomized to direct-mail and opt-in self-sampling groups was 28 and 41 days, respectively (57). Thus, vaginal self-samples collected in a non-clinical setting are anticipated to remain stable in a real-world cervical cancer program. More comprehensive analytical studies like the one described above are needed for other self-sample devices and hrHPV assays to optimize preanalytical workflows and rejection criteria for hrHPV self-sampling.

## CERVICAL CYTOLOGY REMAINS VALUABLE FOR TRIAGE AND REFLEX TESTING FOLLOWING HPV PRIMARY SCREENING

Triage or reflex testing plays a critical role in cervical cancer screening by distinguishing between transient, harmless hrHPV infections and those that may progress to precancer or cancer. Since most hrHPV infections resolve without intervention, triage testing helps identify women truly at risk. FDA-approved methods to determine appropriate triage include HPV genotyping, cytology, and p16/Ki-67 dual stain testing to stratify risk by distinguishing hrHPV genotypes or cellular changes indicative of disease progression (63, 64). In this context, cytology will remain clinically useful; however, when the cytopathologist is aware of the patient’s HPV status, it introduces a bias whereby hrHPV-negative people are more likely to be categorized as negative for intraepithelial lesions, and hrHPV-positive people are more likely to be categorized as having atypical morphology, limiting the rationale for continuing co-testing (65). Triage testing allows for targeted follow-up, reducing unnecessary procedures and improving overall risk management. As a result, triage testing supports more personalized care while minimizing psychological and physical harms associated with overtreatment. Evaluation for the best follow-up triage after hrHPV is detected with primary HPV screening is still underway.

## CHALLENGES ADOPTING HPV PRIMARY SCREENING

Transitioning from the traditional Pap test-based cervical cancer screening to primary HPV testing represents a paradigm shift in preventive healthcare. While primary HPV testing offers superior sensitivity in detecting hrHPV—the principal causative agents of cervical cancer—it also presents significant implementation challenges to patients, providers, and institutions that must be addressed to ensure its widespread adoption and effectiveness.

For nearly a century, the Pap test was the cornerstone of cervical cancer prevention, significantly reducing incidence and mortality rates (1). As a result, providers and patients alike have developed a strong reliance on Pap-based screening and remain skeptical about any change to this time-honored practice (3). Many patients do not understand the role of HPV in the development of cervical cancer, are confused about the differences between HPV and Pap tests, and are uneasy about extending screening intervals. Moreover, those with prior abnormal cytology are worried about their personal risk of transitioning to HPV-only screening. Additionally, there is confusion regarding the distinction between cervical cancer screening and STI screening. Clear communication, in-person discussions between patients and their providers, and accessible educational materials are essential to improve understanding and acceptance (66).

Clinical providers recognize the potential benefits of primary HPV screening but remain concerned about the lack of consensus, guideline variability, extended screening intervals, changes to patient management, reimbursement, and potential unintended consequences (67). Although a concern, HPV-negative, Pap-positive results are relatively uncommon, occurring in about 3%–5% of co-testing cases in women aged 30 and older, and the likelihood of detecting high-grade lesions in this group is low; most cases involve low-grade cytologic abnormalities (68–71). These discordant results may arise from false-positive Pap findings, false-negative HPV tests, or non-HPV-related abnormalities like inflammation or atrophy (72, 73). In a study of 4 million Pap tests, there were 54 cases of HPV-negative, Pap-positive cervical cancer; only 18 cases were asymptomatic and clinically undetectable at the time of diagnosis (71). Providers also share significant concerns regarding the challenge of patient education. Combined, it becomes clear why providers may be resistant to adopting primary HPV screening.

Healthcare system limitations also hinder implementation (74). Reimbursement inconsistencies across Medicare, Medicaid, and private insurance create access barriers, particularly for marginalized populations. A fragmented healthcare system, inadequate patient tracking, and limited data on HPV screening uptake further complicate efforts. Technical barriers, such as EHR system limitations and unclear billing codes, add complexity to integrating HPV screening into routine care. Moreover, a significant number of laboratories are not equipped with HPV testing platforms that are FDA approved for primary screening, delaying widespread adoption. Some view the transition as politically or financially motivated.

Approximately 20% of cervical cancer screenings in the United States still rely on Pap tests alone, particularly among lower-income and minority populations who often receive care in under-resourced settings (4, 66). These groups face barriers such as a lack of insurance coverage, limited access to HPV testing facilities, and distrust of changes in medical recommendations. Ensuring equitable access to HPV self-sampling could help reach individuals who are historically underscreened, yet the logistics of implementing and providing follow-up care for this approach remain unresolved, and regulatory clearance for remote self-collection remains forthcoming.

The move to primary HPV screening represents a necessary and evidence-based evolution in cervical cancer prevention (75). However, significant challenges—including provider resistance, laboratory infrastructure updates, insurance and reimbursement issues, and patient education—must be systematically addressed. Through coordinated efforts among healthcare professionals, policymakers, and patient advocacy groups, the transition can be achieved in a way that maintains trust, minimizes disparities, and optimizes cervical cancer prevention efforts.
